# Pattern Analysis of Oxygen Saturation Variability in Healthy Individuals: Entropy of Pulse Oximetry Signals Carries Information about Mean Oxygen Saturation

**DOI:** 10.3389/fphys.2017.00555

**Published:** 2017-08-02

**Authors:** Amar S. Bhogal, Ali R. Mani

**Affiliations:** UCL Division of Medicine, University College London London, United Kingdom

**Keywords:** entropy, fractal, multiscale entropy, oxygen saturation variability, pulse oximetry, SpO_2_

## Abstract

Pulse oximetry is routinely used for monitoring patients' oxygen saturation levels with little regard to the variability of this physiological variable. There are few published studies on oxygen saturation variability (OSV), with none describing the variability and its pattern in a healthy adult population. The aim of this study was to characterize the pattern of OSV using several parameters; the regularity (sample entropy analysis), the self-similarity [detrended fluctuation analysis (DFA)] and the complexity [multiscale entropy (MSE) analysis]. Secondly, to determine if there were any changes that occur with age. The study population consisted of 36 individuals. The “young” population consisted of 20 individuals [Mean (±1 SD) age = 21.0 (±1.36 years)] and the “old” population consisted of 16 individuals [Mean (±1 SD) age = 50.0 (±10.4 years)]. Through DFA analysis, OSV was shown to exhibit fractal-like patterns. The sample entropy revealed the variability to be more regular than heart rate variability and respiratory rate variability. There was also a significant inverse correlation between mean oxygen saturation and sample entropy in healthy individuals. Additionally, the MSE analysis described a complex fluctuation pattern, which was reduced with age (*p* < 0.05). These findings suggest partial “uncoupling” of the cardio-respiratory control system that occurs with aging. Overall, this study has characterized OSV using pre-existing tools. We have showed that entropy analysis of pulse oximetry signals carries information about body oxygenation. This may have the potential to be used in clinical practice to detect differences in diseased patient subsets.

## Introduction

Pulse oximetry is a technique used to measure oxygen saturation (SpO_2_) non-invasively. It is a method commonly used clinically whether that be in intensive care, in surgery, or in some out-patient clinics (Jubran, 2015). The use of pulse oximetry in these settings has helped reduce the need for invasive arterial blood gases analysis and increase the detection of hypoxaemia (Jubran, 2015), as defined as an SpO_2_ value observed as <95% (Amoian et al., 2013).

It has become increasingly recognized for the use of variability analysis in oxygen saturation to further gauge the regulation of blood oxygenation. However, the methods currently used are not as robust as the methods described in other physiological measurements such as heart rate variability (Garde et al., 2014). The benefit of physiological variability analysis is that it can give us useful information on the integrity of physiological control system (Shirazi et al., 2013; Raoufy et al., 2017). SpO_2_ variability analysis has the potential to be used for monitoring the integrity of the cardiorespiratory control system which is involved in tissue oxygenation. Oxygen saturation variability (OSV) has also been well-characterized in pre-term infants, where it was observed that over the postnatal period there is a steady increase in OSV with no change observed in the mean SpO_2_ value (Dipietro et al., 1994). Moreover, OSV has been studied to be used diagnostically. A more recent study (2016) conducted in a tertiary level hospital in Bangladesh investigated whether implementing OSV in a predictor tool could improve admission of critically ill young children (Garde et al., 2016). They found that incorporating methods that quantified OSV improved the sensitivity and specificity of the tool for identifying these children (Garde et al., 2016). In addition, OSV has been used in the diagnosis of sleep disordered breathing whereby the SpO_2_ characterization adequately described the SpO_2_ modulation in order to identify those at risk of sleep disordered breathing (Garde et al., 2014).

The main issue with previous reports on OSV is the characterization of “variability” and the lack of establishment of a “normal variability” in a healthy population. Additionally, the methods used to describe variability in these studies are primarily linear (i.e., standard deviation, Delta 12 s, saturations >2%, etc.), which to some extent miss out the pattern of SpO_2_ fluctuations (Dipietro et al., 1994; Cox et al., 2011; De Jesus et al., 2011; De Oliveira et al., 2012; Amoian et al., 2013; Garde et al., 2014, 2016; Hoffman, 2016). Hence, the initial need for a study examining healthy individuals in an adult population to characterize the OSV with more sophisticated methods for variability analysis. This can then allow the subsequent comparison with patient sub-populations to garner insight into the disease pathophysiology. The tools selected for this study will be measuring the regularity, complexity, and self-similarity of the fluctuations, which have been shown to better understand biological systems (Richman and Moorman, 2000).

There exist indices that describe the pattern and complexity of fluctuations in physiological time-series. For example, sample entropy is a tool to describe regularity in time series and has been well-established in the study of the cardiovascular system dynamics (Richman and Moorman, 2000). Reduction in the sample entropy of a time-series has been shown to reflect the difference between normal and diseased participants, i.e., in patients with sepsis (Ahmad et al., 2009; Gholami et al., 2012), cirrhosis (Mani et al., 2009), and in obstructive sleep apnoea (Al-Angari and Sahakian, 2007).

Multiscale Entropy (MSE) is an extension of sample entropy and has been used as a tool to describe complexity in a time series (Costa et al., 2002). Complexity in this context can be defined as “meaningful structural richness” which incorporate correlations over multiple scales (Costa et al., 2002). This is achieved mathematically by creating several sub-time series from the main series and calculating the sample entropy of each scaled series (Costa et al., 2002). This can then be plotted to show these cross-scale correlations (Costa et al., 2002). This can be applied in a clinical setting to provide extra prognostic information (Watanabe et al., 2015). MSE has been used to analyse many bio-signals (Gao et al., 2015) such as EEG dynamics in Alzheimer's disease (Mizuno et al., 2010) and heart rate variability analysis for predicting hospital mortality (Norris et al., 2008). Entropy is linked to the concept of information content in a given time-series (Mitchell, 2009). Thus, reduced entropy in a physiological setting can be interpreted as reduced information processing or less engagement the component of within a control system (Pincus, 1994).

Many physiological readings exhibit a fractal-like dynamic. Detrended fluctuation analysis (DFA) is a technique that examines scaling and fractal-like behavior in fluctuating time-series (Peng et al., 1995). The method entails measuring the correlations in the series over several scales to determine if they fit a fractal-like pattern. In this type of analysis when something is fractal-like; it has self-similarity. Many physiological rhythms shares this trait, as they gives rise to self-similar fluctuations over different time scales (Goldberger and West, 1987).

The aim of this study is to establish how oxygen saturation varies in a healthy population and what techniques are best suited to quantifying this variability. Furthermore, we wanted to analyse the regularity, complexity, and self-similarity of these fluctuations using the aforementioned tools. Additionally, as other physical parameters such as heart rate variability (Zhang, 2007) differ greatly with age, the study was also set up to determine if there is a significant difference between a young and old population.

## Materials and methods

### Study population

This study was registered and approved by the UCL Ethics committee (10525/001). The study population was made up 36 individuals, which was later split into two groups for further analysis. The “young” population, defined as members under the age of 35, consisted of 20 individuals [9 Men, 11 Women; Mean (±1 SD) age = 21.0 (±1.36 years)]. The “old” population, defined as members aged 35 and over consisted of 16 individuals [8 Men, 8 Women; Mean (±1 SD) age = 50.0 (±10.4 years)]. In order to establish a healthy study population some exclusion criteria were set; which covered Asthma, COPD, Sickle Cell Anaemia, and Pulmonary Fibrosis. Additionally, the smoking status of the participants was recorded for the possibility of retrospective analysis.

### Assessment of oxygen saturation variability

#### Data collection

Each participant was connected to a pulse oximeter connected to an AD convertor (ADInstrument Ltd, Australia). The recording was initially completed over a 1 h period at a sampling rate of 1 k/s as a pulse recording was also taken alongside. However, the resolution was reduced to 1/s for the pulse oximeter using standard desampling protocol (LabChart). The main reason for this is that pulse oximeter readings are not sampled at such a high rate, and thus at that resolution, the variability presented would not reflect true SpO_2_ variation. The data for pulse oximetry was then extracted into an ASCII file for analysis. Prior to analysis the data was visually scanned for artifacts and the artifact was replaced by the mean value of the entire data set using zero-line interpolation. We chose this method as based on previous findings, it was shown to be the most stable when compared to other methods for artifact replacement (Wejer et al., 2014). As we recorded data ourselves, the data was clean thus only a small number of participants were affected by the noise reduction methods. Recordings with more than 5% artifacts were excluded from analysis. The data collected in this study is shared at PhysioNet^1^.

#### Linear and non-linear analysis of OSV

The linear analysis was to first establish general tendencies of the data set. The mean SpO_2_ and standard deviation were calculated for all participants using an *ad-hoc* programme in MATLAB (MathWorks, R2017a). Poincare' plot analysis was used to calculate short-term (SD1) and long-term (SD2) variability. The Poincaré plot is a geometrical technique that provides visualization of variability (Billman, 2011). It is constructed by plotting each signal in a given time-series as a function of the preceding signal (Hsu et al., 2012). The shape of this plot gives information on how the consequent signals are correlated within a time-series. Therefore, it has been used as a method of distinguishing short-term from long-term fluctuations (Mani et al., 2009).

#### Detrended fluctuation analysis

This method quantifies fractal-like correlation properties on the time series (Peng et al., 1995). In this method, the root mean square of fluctuation is calculated in an integrated and detrended dataset, specific to observation windows of particular sizes, and then plotted against this window size on a log-log scale. If the relationship is linear, then the data can be labeled as fractal-like. Furthermore, the slope of this line (α) can be calculated and used a tool for comparison. α = 0.5 indicates uncorrelated random data. An α > 0.5 or ≤1.0 indicates long-range power-law correlations. An α = 1 corresponds to 1/f dynamics. For α > 1, correlations exist but cease to be of a power-law form (Peng et al., 1995). Software shared at PhysioNet was used for Detrended Fluctuation Analysis (DFA; Goldberger et al., 2000).

#### Sample entropy

This index quantifies the degree of randomness vs. the degree of regularity in a time series. It calculates that probability that an event with window length, m, and degree of tolerance, r, will be repeated at later time points (Richman and Moorman, 2000). A low sample entropy is indicative of a regular time series, while a high sample entropy indicates an irregular time series. For this study, the sample entropy was calculated using MATLAB codes shared at PhysioNet (Goldberger et al., 2000) with m set at 2 and *r* at 0.2 (Richman and Moorman, 2000).

#### Multiscale entropy

Multiscale Entropy (MSE) looks at the sample entropy at different scales to determine if there are any correlations. The process creates “coarse-grained” time series which are produced by averaging the time points within a given window of increasing length, Γ (Costa et al., 2002). The sample entropy is then created for each of these time series and then plotted against scale (with window size of m and tolerance of r for the sample entropy; Costa et al., 2002). A constant MSE graph reflects a complex time series (e.g., 1/f dynamics), while should the values decrease as the scale increases, then the complexity is low (e.g., uncorrelated random noise; Costa et al., 2002). Little is known about the opposite correlation. MSE was calculated using MATLAB codes shared at PhysioNet (Goldberger et al., 2000; Richman and Moorman, 2000).

#### Statistical analysis

Initially, sample size was calculated based on the calculated differences from the MSE data in the pilot study run in December 2016^2^. This value was calculated at 20 participants per group to reach a significant difference in MSE between young and old population (error type I = 0.05, power: 90%), however, because statistical significance was reached at ~18 participants per group we stopped recruiting. For the comparison between the two participant populations several statistical tests were used, utilizing PRISM 7 and SPSS software. The two-tailed Student's *t*-test was used for testing the effect of age on the alpha value from the DFA analysis, the sample entropy, the standard deviation, and the mean SpO_2_. Lastly, a two-way ANOVA analysis was used to test the effect of age on the MSE values.

## Results

### Participants

No participant recruited chose to withdraw from the study so all participants were considered for analysis. However, upon partial medical history, one participant was excluded from the study due to categorization under the exclusion criteria.

### The pattern of oxygen saturation variability in the participant population

#### Variability analysis

**Figure 1:**
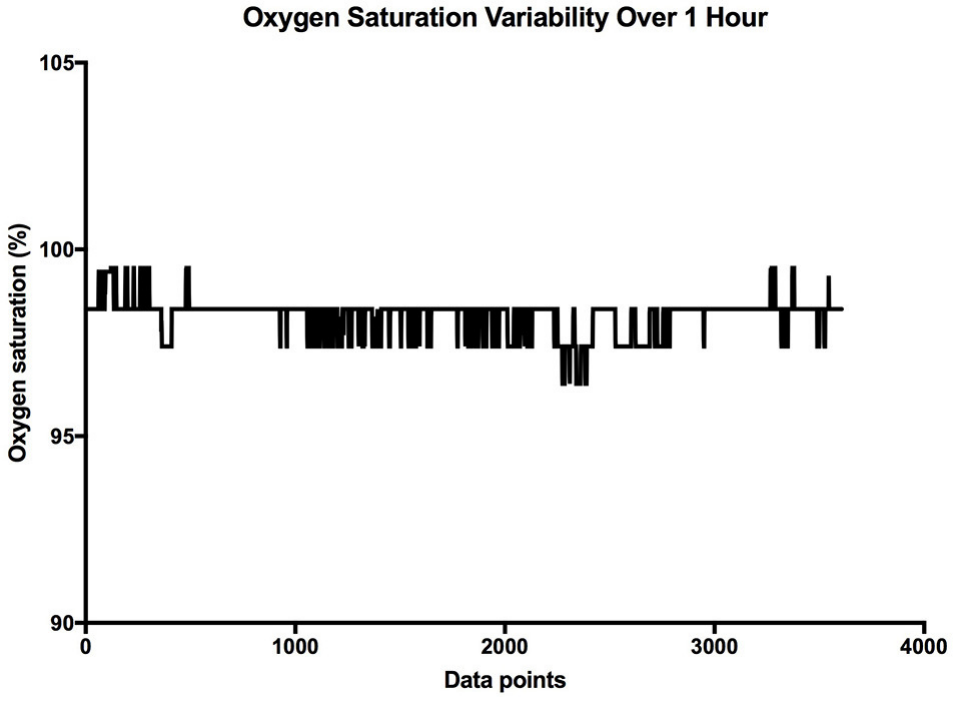
Sample SpO_2_ data collected over 1 h. The X axis is the cumulative data points, and the Y axis is the oxygen saturation.

**Figure 2:**
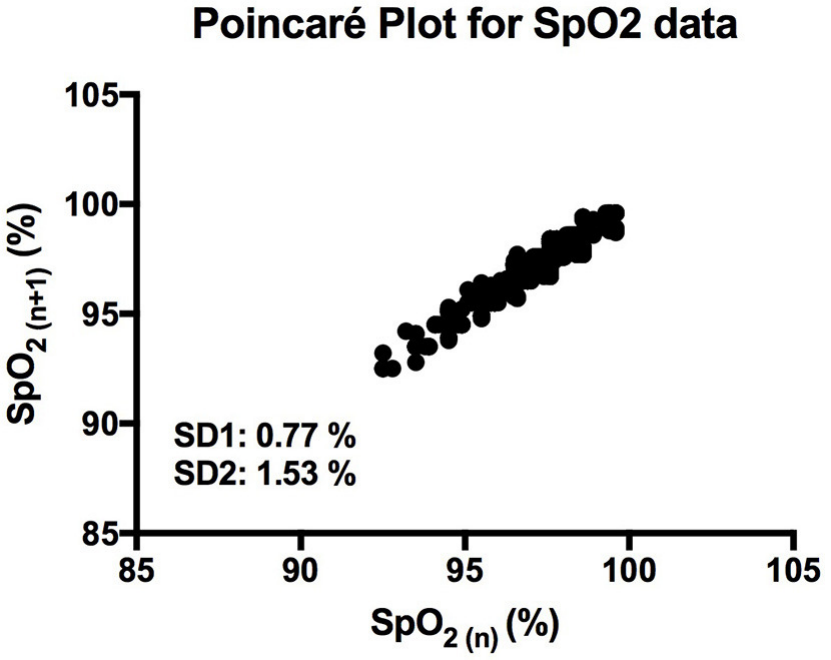
Poincaré plot showing the correlation between consecutive SpO_2_ readings in a representative participant. SD1 and SD2 represent the length and width of the plot across the line of identity.

**Figure 3:**
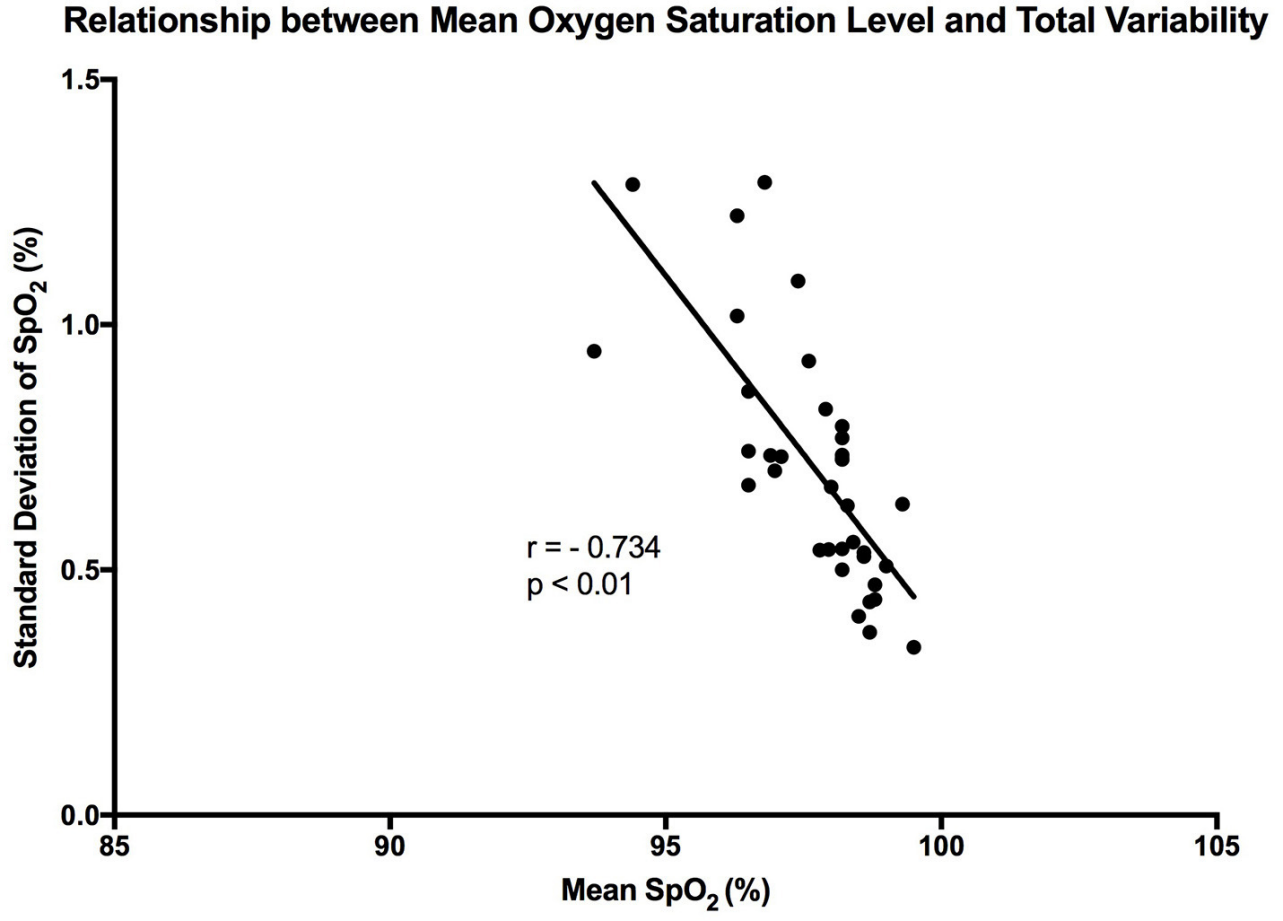
Graph showing the linear trend that exists between mean SpO_2_ level and total variability. Each point represents a participant in the study. “r” represent the Pearson correlation coefficient.

It is clear that over 1 h, the oxygen saturation readings exhibit fluctuations (Figure 1). This variability is mixed with desaturation events and saturation events. The descriptive statistics presented in Table 1, show that the mean SpO_2_ for the population studied is 97.7%. In regards to the variability, the mean standard deviation for the pulse oximetry recording was 0.707%, showing overall a small degree of variability. Using Poincare' plot, we can see that overall there was higher variability across the line of identity (SD2 = 0.987 vs. SD1 = 0.500). This indicates that the variability was predominantly made up of long-term variations (SD2) rather than short term variations (SD1; Figure 2). We also analyzed the relationship between mean SpO_2_ level and total variability (Figure 3), which showed that at higher SpO_2_ levels there is less overall variability (*r* = −0.734, *p* < 0.01).

**Table 1:** Linear and non-linear characteristics of SpO_2_ for the study population.

**Mean SpO_2_ (%)**	**Standard deviation of SpO_2_ (%)**	**SD1 (%)**	**SD2 (%)**	**DFA (α1)**	**DFA (α2)**	**Sample entropy**
97.7 ± 1.25	0.707 ± 0.247	0.500 ± 0.175	0.987 ± 0.351	1.30 ± 0.11	0.87 ± 0.10	0.89 ± 0.35

*Data are expressed as means ± SD. SD1 is the short-term SpO_2_ variability, SD2 is the long-term variability. SpO2 values >95% is considered normal, DFA, Detrended fluctuation analysis. Sample size = 36*.

#### Detrended fluctuation analysis

**Figure 4:**
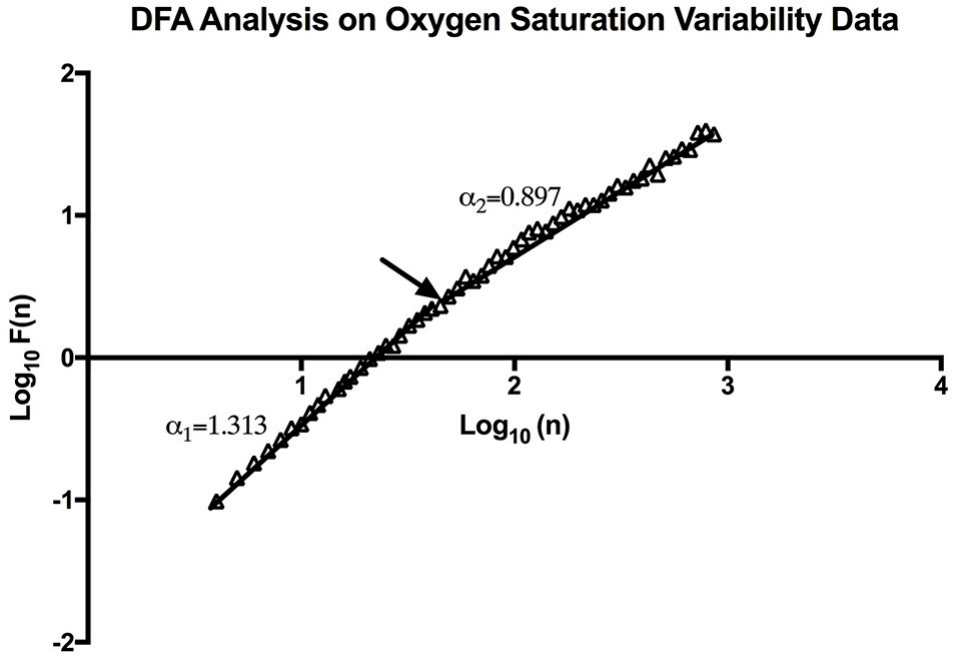
An example of DFA Analysis on SpO_2_ variability data showing the linear trend when plotting (n) and F (n) on a log-log scale. The arrow indicates the approximate point of the cross-over phenomenon.

Through DFA analysis it can be seen that OSV is fractal-like in nature. The result of this analysis can be seen below (Figure 4). Furthermore, the analysis revealed there to be a “crossover phenomenon” something also seen in heart rate variability analysis (Peng et al., 1995). The mean α_1_ score for the participants analyzed is 1.30 (Table 1), and the mean α_2_ score is 0.87 (Table 1). The α_1_ is between pink noise (1/f dynamics) and Brownian noise while the α_2_ is between white noise and pink noise. Thus, this result shows that not only is SpO_2_ variability fractal in nature, but the variation itself is complex in its constitution. The relationship between the α values and mean SpO_2_ level was tested, however, we found no statistically significant correlation between the two.

#### Sample entropy and multiscale entropy

**Figure 5:**
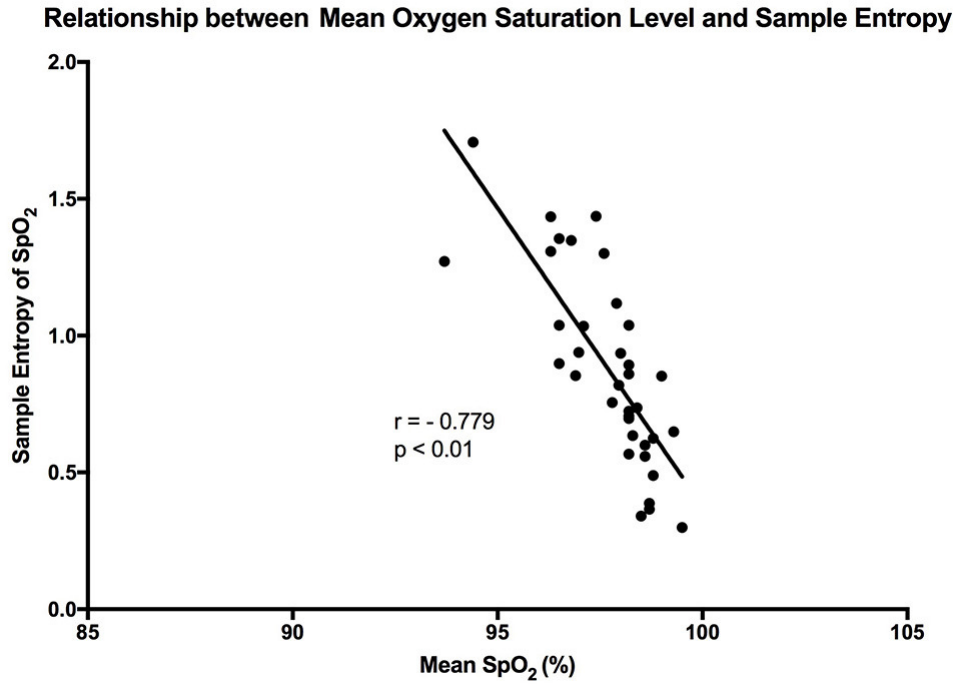
Graph showing the linear relationship that exists between sample entropy and the mean SpO_2_ level. The points are representative of the participants in the study, and “r” is the calculated Pearson correlation coefficient.

**Figure 6:**
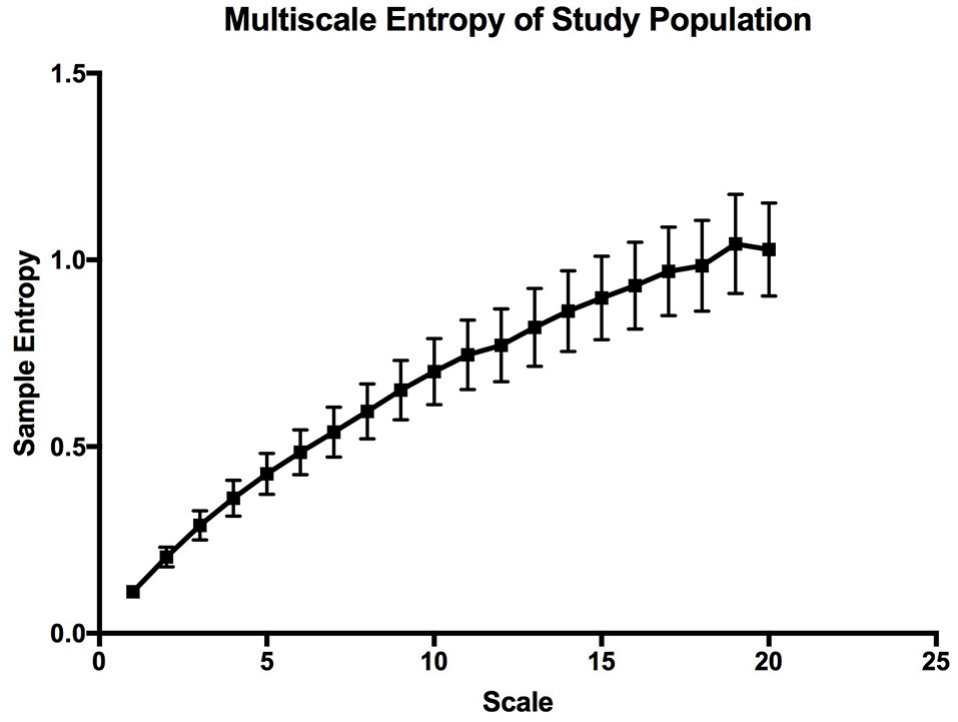
Multiscale entropy graph describing the overall complexity of the whole study population. The error bars are calculated sample error of the mean values.

The mean sample entropy using a window length (m) of 2 was 0.877. The relationship between Sample entropy and the mean SpO_2_ level was also tested (Figure 5), and the graph showed there was a strong linear relationship between the two variables (*r* = −0.779, *p* < 0.01). Showing that a high mean SpO_2_ level is indicative of a more regular pattern of variability. The multiscale entropy (MSE) analysis revealed that the sample entropy increases as the scale increases (Figure 6). There was also a significant inverse correlation between the mean SpO_2_ level and the sum of the MSE values (*r* = −0.66, *p* < 0.001). This indicates that high mean SpO_2_ is associated with decreased complexity of pulse oximetry signals.

### The effect of aging on the variability of oxygen saturation

Following on from the previous results, the effects of aging on the OSV were studied. Statistically there was a significant difference between the mean ages in both groups (*p* < 0.05) allowing for the comparison of the raw data. Firstly, looking at mean SpO_2_ and standard deviation of SpO_2_, there was no significant difference between the two groups (Table 2). The comparison of both α values (DFA) between the groups also revealed no significant difference between the two participant populations. The analysis also revealed that there is no significant change in these parameters between genders (data not shown).

**Table 2:** The effect of age on the measures of SpO_2._

	**Average Age (years)**	**Mean SpO_2_ (%)**	**Standard Deviation of SpO_2_ (%)**	**DFA (α1)**	**DFA (α2)**	**Sample entropy**
Participants < 35	21.1 ± 1.4	98.02 ± 0.81	0.68 ± 0.22	1.28 ± 0.098	0.87 ± 0.11	0.86 ± 0.29
Participants > 35	49.9 ± 10.4	97.31 ± 1.59	0.75 ± 0.28	1.34 ± 0.11	0.88 ± 0.086	0.90 ± 0.42
*p*-value	0.000^*^	0.090	0.408	0.102	0.78	0.739

*Values are displayed in the format of means ± SD. The P-values are calculated using the Student's t-test and significance was set at p < 0.05*.

* *Value < 0.05 as tested by the Mann–Whitney U-test*.

**Figure 7:**
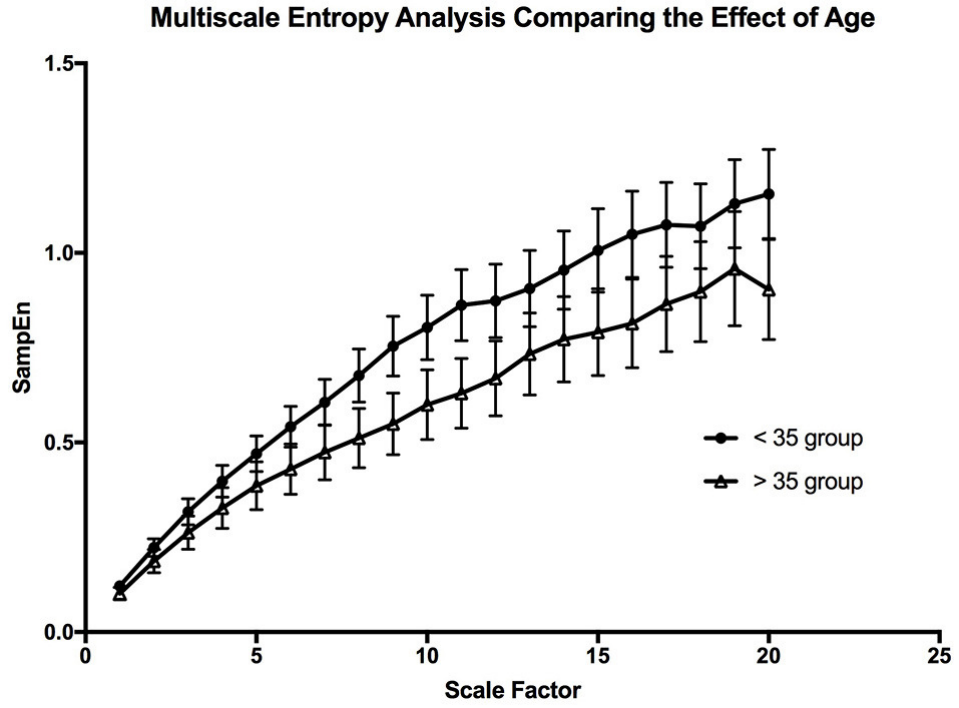
The graph is depicting the effect of aging on the complexity of the oxygen saturation variability. The error bars are the standard error of the mean at each scale, and each point is the mean value at that scale from all the participants in the age group.

The multiscale entropy analysis showed a significant [*F*_Age_ (1, 19) = 99.02; *p* < 0.0001, *F*_Scale_ (19, 19) = 65.44; *p* < 0.0001] reduction in the sample entropy values between both age groups using a two-way ANOVA analysis (Figure 7). The difference is more apparent at the higher scales showing that the reduction in complexity seems to be made more apparent when long-term variations are considered. This reduction in the MSE of OSV seems to be the most sensitive variable affected by aging.

## Discussion

In the present study, we used non-linear dynamics to assess the pattern of SpO_2_ variability. Our results showed for the first time that SpO_2_ exhibited a fractal-like pattern of fluctuation as assessed by DFA. We also showed that the entropy of SpO_2_ can be easily calculated using both sample entropy and multiscale entropy techniques.

### The overall characteristics of OSV

From the raw data, it is clear that OSV is apparent in a healthy population. However, the amount of overall variability changes from person to person. When analysing the pattern of OSV, the time series appears to be regular when compared with other physiological time series (Cuesta-Frau et al., 2009; Hautala et al., 2010; Raoufy et al., 2016). The mean value of sample entropy of R-R interval variability in 5-min recording of adults was close to 1 (Hautala et al., 2010). In respiratory rate variability, the sample entropy of healthy adult men was closer to 1.8 (Raoufy et al., 2016). Thus, from this study it seems that the regularity of OSV (sample entropy = 0.877) is closer to that of heart rate variability (HRV) data (Hautala et al., 2010).

Through Poincaré analysis, the variability was characterized to be more long term rather than short term (SD2 > SD1). Both DFA and MSE analysis uses scaling and it enables us to study oxygen saturation dynamics from both long-term and short-term views. DFA showed that the pattern of OSV is fractal-like. However, the slope seems to show a crossover effect (α_1_ = 1.30, α_2_ = 0.87). On very short scales the fluctuation of SpO_2_ seem to be very stable, thus the higher α_1_ value (Peng et al., 1995). While at larger scales there is more fluctuation, reflecting a more complex behavior. Noticeably though, the α of the SpO_2_ data is similar to the data of heart rate variability in healthy individuals (Peng et al., 1995).

### Association between mean oxygen saturation level and OSV measurements

It has been reported in previous studies that OSV is inversely related to the oxygen saturation level in preterm infants (Dipietro et al., 1994). This was confirmed by our findings, where a strong linear trend existed between the two variables in healthy adults (*r* = −0.734). Essentially, at the higher oxygen saturation levels there was less overall variability. This relationship was stronger in the present study when compared with the results of the aforementioned study (Dipietro et al., 1994). A possible reason behind this is that DiPietro et al. were studying oxygen saturation in preterm infants, whose health may be compromised. Thus, this relationship could be an indication of health, whereby a reduction in the phenomenon could indicate compromise of the cardiorespiratory system. It could also show that the oxygenation regulatory system is not mature enough in preterm infants. Furthermore, the relationship between oxygen saturation and sample entropy was stronger (*r* = −0.779), where there was an increase in sample entropy at lower mean SpO_2_ levels. This could reflect tighter system coupling when oxygen saturation is low. According to Pincus (1994), enhanced coupling of signals within a complex system increases the overall entropy. This essentially means that a higher entropy indicates that all the components of the system are connected and in communication (Pincus, 1994). In order to correct a lower mean oxygen saturation level the system needs to be engaged. This is the advantage of using entropy analysis rather than standard deviation in describing physiological variability. The same inverse relationship was also observed between mean SpO2 and MSE, showing that entropy of pulse oximetry signals carries information about mean oxygen saturation. Entropy analysis of physiological signals has been extensively used in the last two decades. Theoretical studies initially suggested that greater physiological signal regularity indicated increased system isolation (Pincus, 1991, 1994). Thereafter, progress in computational techniques for entropy estimation led to development of approximate entropy, sample entropy and MSE. Application of these entropy indexes in physiological time-series provided evidence for a link between system connectivity and entropy in health and disease. For instance, Gholami et al. (2012) reported that impaired responsiveness of the cardiac pacemaker to cholinergic control may present a decreased sample entropy of the heart rate variability during systemic inflammation. A recent report has also shown that sample entropy (as well as MSE) analysis of body temperature time-series may represent the engagement of thermoregulatory system during systemic illnesses such as chronic liver failure (Garrido et al., 2017). A relationship between mean SpO_2_ and entropy of the pulse oximetry signals is an interesting finding that goes along with these lines of research. However, details of the significance of this relationship, await further investigations.

### The effect of age on OSV

Previous studies have shown that there is a significant change in MSE of heart rate variability with age (Angelini et al., 2007). This is interpreted as a decrease in complexity that occurs with aging (Shiogai et al., 2010). Our result shows a similar relationship in SpO_2_ variability with the use of MSE analysis. The scaled values for the young population was significantly higher than that of the older population. The lower sample entropy values at each scale does not only suggest reduced complexity, but also might suggest increased system isolation, which may reflect partial “uncoupling” of the control system (Buchman, 2002; Gholami et al., 2012). Applying what Pincus stated (1994), a reduced entropy may describe a reduction in the system coupling and increase system isolation. Our result may be indicative of the partial uncoupling of the systems involved in the cardio-respiratory control through aging. Something that could explain the drop in Po_2_ with aging (Pocock et al., 2013). There is a benefit from using variability analysis in describing physiological rhythms. There is a clear limitation in using reductionistic methods to describe physiological systems, as in order to achieve meaningful results we need to disrupt the system (Shirazi et al., 2013). Thus, what you study may not display features of the original system, but rather a perturbed system (Altimiras, 1999). It is only through integrative approaches such as pattern analysis that one can truly characterize the complexities of a system (Altimiras, 1999), in this case the cardio-respiratory control system. Our study indicates that pattern analysis of SpO_2_ variability carries information on the integrity of body oxygenation with potentials to be used in clinical practice. It may also provide a tool to study dynamic interactions of organ systems in the emerging field of network physiology (Bartsch et al., 2015).

### Limitations of the study and future work

A potential limitation of the study is that OSV is affected by activity levels (Dipietro et al., 1994; Garde et al., 2014). During the recording, all participants were sitting down, however, their activities varied from reading to conversing. This may have affected the amount of overall variability. However, as we also used techniques less sensitive to change in activity (Hautala et al., 2010) this effect was minimalized. A larger study is needed to follow up on these results to ensure adequate coverage of a healthy population. This would also be key in comparing the effect of aging on OSV.

Future studies could look at linear and non-linear indices of SpO_2_ variability and whether these indices can be assigned clinical significance in the context of disease, as it has done in other physiological variables (Peng et al., 1995; Ahmad et al., 2009; Donaldson et al., 2012; Raoufy et al., 2016). Additionally, novel techniques could be applied to the raw data of this study population such as memory analysis (Shirazi et al., 2013). This technique can further explain the controllability of oxygen saturation and how past fluctuations impact future fluctuations.

## Conclusion

The present study has established the pattern of OSV in a normal healthy population. The total variability predominantly consists of long term variations, and is dependent on the mean SpO_2_ level. The application of sample entropy analysis and MSE analysis to the data has provided novel information about the regularity and complexity of this variability. The fractal nature of OSV, as provided through DFA analysis suggests that structurally, physiological variables may all share this trait. Furthermore, by investigating the effect of aging on OSV, we have garnered insight into the control of oxygen saturation, and how this control system is impaired with aging.

## Author contributions

The study was Conceptualized by AB and AM. The Data was collected by AB. Formal analysis was conducted by AB. Software was developed by AB and AM. AM supervised the study. AB wrote the initial draft. Reviewing and editing was completed by AM.

### Conflict of interest statement

The authors declare that the research was conducted in the absence of any commercial or financial relationships that could be construed as a potential conflict of interest.

## Footnotes

^1^PhysioNet- https://physionet.org

^2^http://www.stat.ubc.ca/~rollin/stats/ssize/n1.html
